# Examining common mental health disorders in people living with HIV on methadone maintenance therapy in Hanoi, Vietnam

**DOI:** 10.1186/s12954-021-00495-3

**Published:** 2021-04-23

**Authors:** Anisa Y. Mughal, Melissa Ann Stockton, Quynh Bui, Vivian Go, Brian W. Pence, Tran Viet Ha, Bradley N. Gaynes

**Affiliations:** 1 School of Medicine, The University of Pittsburgh, 3550 Terrace Street, Pittsburgh, PA 15213 USA; 2 Epidemiology Department, University of North Carolina At Chapel Hill Gillings School of Global Public Health, 135 Dauer Dr, Chapel Hill, NC 27599 USA; 3 The UNC Vietnam Office, Yen Hoa Health Clinic, Lot E2, Duong Dinh Nghe Street, Yen Hoa Ward, Cau Giay District, Hanoi, Vietnam; 4 Department of Health Behavior, University of North Carolina At Chapel Hill Gillings School of Global Public Health, 135 Dauer Dr, Chapel Hill, NC 27599 USA; 5 Department of Psychiatry, University of North Carolina At Chapel Hill School of Medicine, 333 S Columbia St, Chapel Hill, NC 27516 USA

**Keywords:** Common mental health disorder, Depression, Anxiety, Post-traumatic stress disorder, People living with HIV, Methadone

## Abstract

**Background:**

Injection drug use drives HIV transmission in Southeast Asia, where around a quarter of users are living with HIV. Vietnam developed Methadone Maintenance Therapy (MMT) programs to reduce unsafe drug abuse. Common mental health disorders (CMD), including depression, anxiety and post-traumatic stress disorder (PTSD), can worsen MMT outcomes and are highly prevalent among people living with HIV (PLH). We aimed to characterize HIV and CMD among MMT patients and assess the impact of HIV and CMD on MMT engagement outcomes in Hanoi, Vietnam.

**Methods:**

This cross-sectional study was conducted at an urban MMT clinic in Hanoi. Participants were screened for CMD with the relevant sections of the Mini International Neuropsychiatric Interview (MINI). Tabular comparisons and regression models were used to understand the association of HIV and CMD with substance use and methadone compliance.

**Results:**

Of the 400 MMT participants, 22% were living with HIV, 11% a CMD, 27% reported injection drug use, and 27% reported methadone noncompliance. Around 17% of those with HIV also had a CMD. Reporting non injection and injection drug use were each higher among those with CMD regardless of HIV status. In addition, reporting any drug use was much higher among those with both HIV and CMD than among those with neither (73% vs 31%, *p* value 0.001). While methadone noncompliance was lower among PLH than among those without HIV (16.3% vs 30.1%, *p* value 0.010), noncompliance was higher among those with CMD than among those without (40.5% vs 25.6%, *p* value 0.045). Among those without HIV, noncompliance was higher among those with CMD than among those without, but among those with HIV, the opposite relationship was observed.

**Conclusion:**

There is complex overlap between substance use and methadone noncompliance among MMT patients living with HIV, CMD or both. In this population, we found a high prevalence of CMD and substance use among PLH, and a high prevalence of substance use and methadone noncompliance among those with CMD. Prioritizing provision of mental health care services to MMT patients living with HIV can help improve engagement with substance use disorder treatment and reduce the risk of HIV transmission.

## Background

The prevalence of injection drug use globally is estimated to be over 15 million persons [1], who are considered high risk for viral transmission of infectious diseases. The global prevalence of human immunodeficiency virus (HIV) in people who inject drugs (PWID) is estimated to be between 10.8 and 24.8% with the highest number of PWID present in East and Southeast Asia [1]. In Vietnam it is believed that HIV is primarily transmitted through injecting drug use where an estimated 30% of people living with HIV (PLH) also inject drugs; this has major implications for forward transmission of HIV in Vietnam through unprotected sex, groups injecting, and needle sharing practices [2–6].

Methadone maintenance therapy (MMT) has been implemented globally as a way to reduce injecting drug use, minimize withdrawal symptoms and treat opiate dependency [3]. Many countries have implemented MMT programs to reduce work-loss productivity and absenteeism, reduce morbidity and mortality related to substance use and to prevent viral transmission [2]. Vietnam began offering MMT programs in 2008 [2]. Since then, nearly 300 clinics offering MMT across every province in Vietnam have been established [7]. To enroll in MMT, patients must have an opioid dependence, have a stable living condition and be able to afford the daily treatment cost (between 90 and 250 USD per year, depending on dose) [7–9]. Over a decade of experience providing MMT services suggest that streamlining antiretroviral therapy (ART) and MMT programs is both feasible and may present an opportunity to effectively respond to both pandemics in Vietnam [10].

Common mental health disorders (CMD) including depression, anxiety and post-traumatic stress disorder (PTSD) are highly prevalent among MMT patients and among PLH globally [11–13]. CMDs are a leading cause of disability among PLH with a prevalence of over 30% in some low- and middle- income countries (LMICs) [14, 15]. HIV itself imposes a high psychological burden and co-occurrence of HIV and CMDs can lead to reduced adherence to HIV treatment and adverse outcomes, further exacerbating both conditions and reducing daily function [14]. True prevalence of CMDs among PLH is difficult to determine due to limited CMD detection and reporting. Such detection is complicated in LMICs by limited mental health care infrastructure and human resources [16, 17].

However, there are a limited number of studies describing HIV or CMD among MMT patients in Vietnam or Southeast Asia. A previous qualitative study in Vietnam found PWID also living with HIV place importance on mental health care, but face substantial social, physical and economic barriers to already limited existing mental health care services [18]. Other studies in Vietnam have examined CMD among PLH, documenting a prevalence of CMD as high as 43.5%, but have not specifically studied the subset of PWID living with HIV [19]. Regionally, studies in China have examined CMD among MMT patients with only a limited focus on the prevalence of HIV [20].

The co-occurrence of injecting drug use, CMD, and HIV may increase the likelihood for noncompliance to MMT, further exacerbating all three conditions [18, 21]. Therefore, we aim here to describe the prevalence of HIV and CMD among a population of MMT patients and assess the association of CMD and HIV with MMT engagement outcomes, including concurrent substance use and methadone noncompliance.

## Methods

### Study design

Methods of this study have been previously published [22]. Briefly, an intake survey and the depression, anxiety and PTSD sections of a Vietnamese version of the Mini International Neuropsychiatric Interview (MINI) for the Diagnostic and Statistical Manual of Mental Disorders-5 (DSM-5) were administered to a consecutive sample of MMT patients at an urban MMT clinic in Hanoi, Vietnam where at the time 420 patients were receiving daily MMT. Patients were approached by MMT staff while accessing MMT care to solicit interest in participation. Interested patients underwent a verbal consent process as requested by the institutional review board (IRB) to maximize confidentiality. Interviews were conducted with consenting participants on the same day or scheduled within the following week. Interviews were conducted by trained research staff in a private room. Each participant answered a set of demographic, stigma and substance use questions. Patients were compensated 100,000 VND (approximately USD $4.25) for their participation.

### Measures

Diagnoses of depression (major depressive disorder), anxiety (generalized anxiety disorder), and PTSD were identified using the MINI for the DSM-5. Suicidality was defined as a positive response to either of the following two questions*: “*Did you repeatedly think about death (excluding fear of dying), or have any thoughts of killing yourself, or have any intent or plan to kill yourself? Did you attempt suicide?” This question was only asked of individuals who reported either depressed mood or anhedonia on the first two questions of the MINI module for Major Depressive Episode.

Hazardous alcohol use was defined as having had three or more alcoholic drinks within a three-hour period at least three times in the last three months. Any non-injection drug use in the last three months was defined as using any of the following drugs: Heroin, opium, ecstasy or MDMA, Marijuana, Xen/Seduzen (benzodiazepine), Methamphetamine, or Promethazine. Any injection drug use in the last three months was defined as injecting drugs on at least one day in the past three months. Participants who reported missing at least one dose of methadone in the last month were considered non-compliant. HIV status was self-reported.

### Analysis

We completed unadjusted, tabular comparisons of the substance use and methadone noncompliance outcomes by both CMD and HIV status and by CMD status within strata of HIV status. Linear-binomial models were used to estimate the adjusted prevalence difference (aPD) of reported substance use and methadone noncompliance outcomes between three HIV/CMD groups—1) those without HIV, but with CMD 2) those with HIV, but without CMD and 3) those with both HIV and CMD—compared to the referent group with neither HIV nor CMD. All analyses were conducted using STATA.

## Results

### Prevalence of CMD among PLH on MMT

Of the 420 patients receiving MMT at the study site, 400 MMT patients enrolled in this study. The prevalence of HIV among the study population was 21.8% (*n* = 87) (Table 1). The prevalence of every measured mental disorder—depression, suicidality, generalized anxiety disorder and PTSD—was higher among PLH than among those without HIV, according to the MINI criteria. Similarly, the prevalence of any CMD, defined as one or more disorder, was significantly higher among PLH (17.2%) than among those without HIV (9.3%). The average methadone dose was 82.9 mg (range 5–400 mg). On average, PLH were prescribed a significantly higher methadone dose (mean = 156.2 mg) than those without HIV (62.8 mg). Around 80% of both those with and without HIV had been in MMT for at least a year. All PLH were male, 95.4% (*n* = 83) were on ART, 66.7% (*n* = 58) were married or partnered, 34.5% (*n* = 30) had completed some high school and 77% (*n* = 67) were working at least part-time.

**Table 1:** Study participants stratified by HIV status

*n* (%) or mean (SD)	Participants living		*p* value*
	Without HIV (313)	With HIV (*n* = 87)	
Any CMD	29 (9.3%)	15 (17.2%)	**0.051**
Depression	27 (8.6%)	15 (17.2%)	**0.029**
Suicidality	3 (1.0%)	9 (10.3%)	**0.027**
Anxiety	10 (3.2%)	6 (6.9%)	0.072
PTSD	4 (1.3%)	4 (4.6%)	0.127
In MMT care for			0.642
< 1 Year	57 (18.3%)	18 (20.7%)	
≥ 1 Year	255 (81.7%)	69 (79.3%)	
Methadone dose in mg (range 5–400)	62.8 (36.6)	156.2 (95.8)	< .001
Age (range 23–63)	41.2 (7.6)	41.6 (5.7)	0.616
Sex at birth			> .99
Male	310 (99%)	87 (100%)	
Female	3 (1%)	0 (0%)	
Marital status			0.268
Single	55 (17.6%)	20 (23%)	
Married or partnered	235 (75.1%)	58 (66.7%)	
Widowed/separated	23 (7.3%)	9 (10.3%)	
Education			0.641
None	2 (0.6%)	0 (0%)	
Some primary	15 (4.8%)	6 (6.9%)	
Some secondary	116 (37.1%)	30 (34.5%)	
Some high school	148 (47.3%)	46 (52.9%)	
Some technical training	7 (2.2%)	0 (0%)	
Some college	25 (8%)	5 (5.7%)	
Employment			0.205
Working at least part-time	262 (83.7%)	67 (77%)	
Unemployed	45 (14.4%)	19 (21.8%)	
Retired	6 (1.9%)	1 (1.1%)	

Bolding indicates statistical signficicant

*Fisher's exact for binary or categorical variables or *t* test for continuous variables

### Prevalence of substance use by HIV status and CMD

A larger proportion of PLH reported any non-injection (24.1% vs. 18.2%, *p* value 0.223) and any injection drug use (34.5% vs. 24.3%, *p* value 0.074) compared to those without HIV (Table 2). However, PLH had a statistically significant lower prevalence of hazardous alcohol use (20.7% vs 33.9%, *p* value 0.019) compared to those without HIV. Though not shown, a significantly larger proportion of the 44 participants with any CMD reported any non-injection (45.5% vs. 16.3%, *p* value < 0.001) and any injection drug use (50.0% vs. 23.6%, *p* value < 0.001) compared to the proportion among the 356 participants without CMD. As seen in Table 3, within strata of HIV status, larger proportions of those with any CMD reported hazardous alcohol use, any non-injecting drug use, and any injection drug use than of those with no CMD, noting the small sample sizes.

**Table 2:** Substance use in the past three months and Methadone compliance by HIV status

*n* (%) or mean (SD)	Participants living		*p* value*
	without HIV (313)	with HIV (*n* = 87)	
Hazardous alcohol use	106 (33.9)	18 (20.7)	**0.019**
Any non-injection drug use	57 (18.2)	21 (24.1)	0.223
Any injection drug use	76 (24.3)	30 (34.5)	0.074
Missed a dose^♦^	94 (30.1)	14 (16.3)	**0.010**
Avg. number missed^ǂ^ (range 1–12)	2.1 (2.1)	1.3 (0.6)	0.253

Bolding indicates statistical signficicant

*Fisher's Exact for binary variables or *t* test for continuous variables

^♦^Refused to answer *n* = 2

^ǂ^Among whose who reported missing a dose in the last month

**Table 3:** Substance use in the past three months by CMD within strata of HIV status

*n* (%)	Without HIV			With HIV		
	No CMD (*n* = 284)	CMD (*n* = 29)	*p* value*	No CMD (*n* = 72)	CMD (*n* = 15)	*p* value*
Hazardous						
Alcohol use	96 (33.8)	10 (34.5)	> 0.999	12 (16.7)	6 (40.0)	0.074
Any non-injection						
Drug use	43 (15.1)	14 (48.3)	** < 0.001**	15 (20.8)	6 (40.0)	0.181
Any injection						
Drug use	62 (21.8)	14 (48.3)	**0.003**	22 (30.6)	8 (53.3)	0.134

Bolding indicates statistical signficicant

**Fig. 1:**
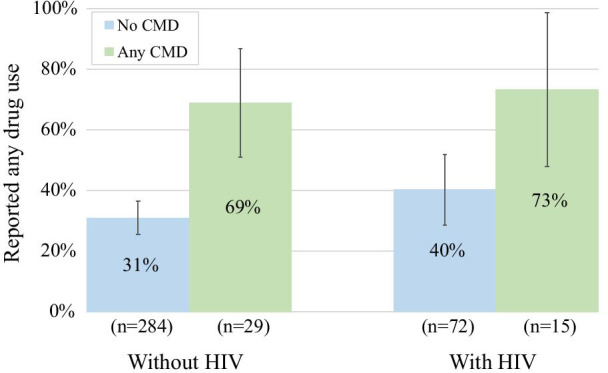
Prevalence reporting any drug use by strata of HIV status and CMD. This figure shows the prevalence of reporting any drug use by strata of HIV status and CMD

As shown in Fig. 1, the prevalence of reporting any drug use (either injection or non-injection) in the last 3 months was higher among those with any CMD (shown in green) than without CMD (shown in blue) for both those living with and without HIV (on the right and left, respectively) (Fig. 1). Further, 73% (SE: 25%) of those living with HIV and any CMD reported any drug use in the last three months whereas only 31% (SE: 5%) of those living without HIV and no CMD reported any drug use in the last three months.

### Methadone noncompliance by HIV status and CMD

PLH had significantly higher methadone doses than those without HIV (156.2 mg vs 62.8 mg, *p* value < 0.001) (Table 1), but a lower prevalence of noncompliance within the previous month (16.3% vs 30.1%, *p* value 0.010) (Table 2). Though not shown, those with CMD had a significantly higher methadone dose (101.9 mg vs 80.5 mg, *p* value 0.046) and a significantly higher prevalence of noncompliance (40.5% vs 25.6%, *p* value 0.045) than those without CMD. As seen in Table 4, among participants without HIV, a larger proportion of those with CMD reported missing a methadone dose in the last month. In contrast, among PLH a smaller proportion of those reporting CMD reported missing a methadone dose in the last month than among those without CMD, although the cell sizes are quite small.

**Table 4:** Methadone compliance by CMD within strata of HIV status

*n* (%) or mean (SD)	Without HIV			With HIV		
	No CMD (*n* = 284)	CMD (*n* = 29)	*p* value*	No CMD (*n* = 72)	CMD (*n* = 15)	*p* value*
Missed a dose^♦^	78 (27.5)	16 (57.1)	**0.002**	13 (18.1)	1 (7.1)	0.450
Avg. number missed^ǂ^ (range 1–12)	2.2 (2.2)	1.9 (1.4)	0.692	1.3 (0.6)	1 (0)	–

Bolding indicates statistical signficicant

*Fisher's exact for binary variables or *t* test for continuous variables

^♦^Refused to answer *n* = 2

^ǂ^Among whose who reported missing a dose in the last month

### Association of HIV and CMD with substance use and methadone noncompliance

**Fig. 2:**
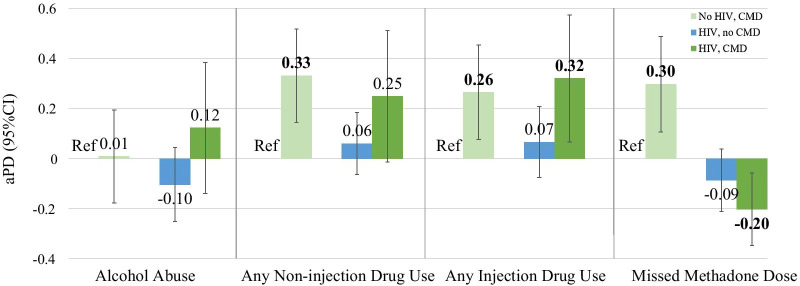
Estimates of Association between HIV status and any CMD on outcomes. This figure shows the adjusted prevalence difference (aPD) for each group compared to the referrent group without HIV or CMD. Prevalence differences for each group compared to the referent No HIV, no CMD group are adjusted for methadone dose

All prevalence differences are adjusted for methadone dose. Figure 2 shows the adjusted estimated prevalence differences by group (no HIV, CMD; HIV, no CMD; HIV, CMD) compared to the referent group (no HIV, no CMD) for each outcome. Reporting non-injection and injection drug use were each higher among those with CMD (shown in light and dark green) regardless of HIV status. *For non-injection drug use*, among those without HIV, the adjusted prevalence of reporting any non-injection drug use was significantly higher among those with CMD (light green) than among the referent group, aPD 0.33(95%CI:0.14, 0.52). *For injection drug use*, among those without HIV, the adjusted prevalence of reporting any injection drug use were significantly higher among those with CMD (light green) than among the referent group, aPD 0.26 (95%CI:0.08, 0.45). Among those with both HIV and CMD (dark green), the adjusted prevalence of reporting any injection drug use were significantly higher than among the referent group, aPD 0.32 (95%CI: 0.07, 0.57). *For methadone noncompliance,* among those without HIV, the adjusted prevalence of reporting a missed methadone dose in the last month was higher among those with CMD (light green) than those without, aPD 0.30(95%CI:0.11, 0.49). However, among those with both HIV and CMD, the opposite relationship was observed (dark green), aPD -0.20(95%CI:-0.35, -0.06). For all outcomes, no large differences were observed between those without HIV or CMD and those with HIV, but without CMD.

## Discussion

Our cross-sectional study aimed to describe the prevalence of HIV and CMD among a population of MMT patients and to assess the association of HIV and CMD with concurrent substance use and methadone noncompliance. We not only found a high prevalence of HIV, but also, among the PLH, a high prevalence of CMD. Concurrent injection and non-injection drug use was higher among PLH than among those without HIV, a trend also evident for those with CMD compared to those without CMD. In fact, the prevalence of any drug use was much higher among those with both HIV and CMD compared to those with neither. While methadone noncompliance was also higher among those with CMD than among those without CMD, noncompliance was actually lower among PLH than among those without HIV. These findings highlight generally worse reported MMT engagement among MMT patients with HIV, with CMD, or with both.

In Vietnam, the HIV epidemic is concentrated in vulnerable or key populations, particularly people who inject drugs. In our sample of MMT patients, nearly a quarter self-reported they were living with HIV. Previous studies of MMT patients in Vietnam have reported a lower prevalence of HIV within MMT clinics ranging from 3.7 to 17.4% [23, 24]. Of our PLH participants, nearly all (95.4%) were on ART and received HIV treatment separately at the same clinic. These findings support research documenting the co-occurrence of substance use disorders and HIV in Vietnam [25].

Research in Southeast Asia has documented a high prevalence of common mental disorders, particularly depression, both among PLH and among patients in MMT. In our study, the prevalence of depression, anxiety and PTSD were at least twice as high among the PLH as among HIV-negative patients; further, the prevalence of suicidality was almost ten times higher among PLH than among HIV-negative patients. In Vietnam, studies have found that 20–36% of PLH reported depression using the Center for Epidemiological Studies-Depression (CES-D) [26–29]. Other countries in this region large injection drug using populations such as China have estimated the prevalence of depression to be upwards of 38% among MMT patients [30]. Another MMT study in the city of Hai Phong found 12.2% prevalence of depression using the MINI [31], which is similar to the total prevalence among our study population. Of note, a study in Northern Vietnam only found 4% of MMT patients reported mild-extremely severe depressive symptoms using the Depression, Anxiety and Stress Scale-21 (DASS-21), though only 3% of their study population had HIV. While limited research has examined anxiety and PTSD among PLH on MMT in Vietnam [32], there is a clear need to address the mental health needs of PLH in MMT.

The relationship between HIV status, CMD, methadone dosing and methadone compliance is complex. In our sample, PLH had significantly higher doses of methadone compared to those without HIV, as did those with CMD compared to no CMD. Previous studies have reported an association between methadone doses above 100 mg/day and a higher prevalence of major depressive disorder [33, 34]. This is also consistent with our sample population as PLH an average methadone dose above 100 mg/day and a higher prevalence of depression than those without HIV. We found that a larger proportion of participants with CMD reported missing a methadone dose than those without. Another study in Vietnam also found anxiety and depression were risk factors for missing methadone doses and suboptimal methadone adherence among MMT patients in Vietnam [35]. Interestingly, our study found that PLH reported a lower prevalence of methadone noncompliance (16.3%) than those without HIV (30.1%, respectively). These findings differ from previous findings that show an association between an HIV diagnosis and reduced compliance among MMT patients [36]. This may be because PLH in our study received HIV care and MMT from the same clinic, simplifying treatment to a singular site. Such an integrated care strategy should be evaluated for its potential to increase methadone compliance.

The HIV epidemic in Vietnam is largely driven by injection drug use and controlling the epidemic will require reducing this risk behavior. We found a high prevalence of injection drug use among both PLH (34.5%) and among those with any CMD (50%). Over half of those with both HIV and CMD reported injection drug use. Other studies of MMT patients in Vietnam found an estimated prevalence of concurrent injection drug use ranging from 11 to 14%, though drug use is believed to vary depending on the length of time since initiation of MMT [23, 37, 38]. Another study found that 54.9% newly admitted MMT patients reported injection heroin use, though this prevalence fell to 15.4% after 24 consecutive months of treatment [39]. Of note, concurrent use of heroin is associated with MMT discontinuation [40] which may lead to an artificially low prevalence of concurrent substance use. Existing studies of MMT clinics in Vietnam have also reported an increased risk of concurrent heroin usage for patients with methadone doses over 100 mg [39]. This suggests that higher doses of methadone are associated with a higher prevalence of concurrent drug use which is consistent with our study population and the higher average methadone dose among PLH and those with CMD. Our findings suggest a need to prioritize addressing the risk of concurrent injection drug use among MMT patients with HIV and CMD.

### Limitations

Our study was limited by the small sample size and limited power to detect significant differences. While nearly a fifth of the 87 PLH sample had any CMD, this amounted to only 15 participants with both HIV and CMD. Further, HIV status was self-reported and we used a strict definition of noncompliance, only allowing for one missed dose in the previous month. However small, this analysis highlights the needs of a particularly vulnerable population of MMT patients burdened by two chronic conditions. We lacked access to more robust mental health and HIV clinical data and did not capture potentially important confounders such as other comorbidities and homelessness. While we did not differentiate between primary and secondary depression, we did use the MINI to determine the prevalence of CMD. The interviewers were blinded to the MINI results before verbally administering the demographic questionnaire and vice versa. We also could not ascertain ART adherence. Due to the cross-sectional nature of the study, the prevalence of concurrent substance use was not tracked over time. While concurrent substance use is expected to change over time, the majority (81%) of patients studied had been in MMT for over a year.

## Conclusion

This cross-sectional study documents the syndemic of HIV, substance use, and mental health disorders among MMT patients in Vietnam where the relationship between substance use behaviors and methadone compliance among PLH with CMD on MMT is complex. However, as Vietnam continues to expand MMT and integrate MMT and HIV care [41–43], prioritizing the mental health needs of this doubly vulnerable population is warranted and could lead to improved treatment outcomes and a reduction in risky behaviors. Integrated care programs will need to account for social, physical and economic barriers to care, such as patient perceptions of stigma towards HIV, injection drug use and limited availability and affordability of mental health care services [41, 43, 44]. Our study adds to the paucity of evidence in Vietnam investigating the overlap of mental health disorders, HIV, and concurrent drug use among methadone maintenance patients; argues for the importance of identifying CMDs in this at-risk population; and highlights the need to develop multifaceted interventions that can improve outcomes for this vulnerable population. Future research should study how the prevalence of CMD, concurrent substance use and methadone dose vary upon enrollment and over time with continued treatment. Results could better inform studies integrating HIV, substance use disorder and mental health treatment.

## Data Availability

The datasets used and/or analysed during the current study are available from the corresponding author on reasonable request.

## Abbreviations

ART: antiretroviral therapy
CES-D: center for epidemiological studies-depression
CMD: common mental health disorders
DASS-21: depression, anxiety and stress scale-21
DSM-5: diagnostic and statistical manual of mental disorders-5
HIV: human immunodeficiency virus
IRB: institutional review board
LMICs: low- and middle-income countries
MINI: mini international neuropsychiatric interview
MMT: methadone maintenance therapy
PLH: people living with HIV
PTSD: post-traumatic stress disorder
PWID: people who inject drugs
